# Augmentation of CD134 (OX40)-dependent NK anti-tumour activity is dependent on antibody cross-linking

**DOI:** 10.1038/s41598-018-20656-y

**Published:** 2018-02-02

**Authors:** Anna H. Turaj, Kerry L. Cox, Christine A. Penfold, Ruth R. French, C. Ian Mockridge, Jane E. Willoughby, Alison L. Tutt, Jordana Griffiths, Peter W. M. Johnson, Martin J. Glennie, Ronald Levy, Mark S. Cragg, Sean H. Lim

**Affiliations:** 1 Antibody and Vaccine Group, Cancer Sciences Unit, Faculty of Medicine, University of Southampton, Southampton General Hospital, Southampton, SO16 6YD UK; 2 Cancer Research UK Centre, Faculty of Medicine, University of Southampton, Southampton General Hospital, Southampton, SO16 6YD UK; 3 Department of Medicine, Division of Oncology, Stanford University, Stanford, CA USA

## Abstract

CD134 (OX40) is a member of the tumour necrosis factor receptor superfamily (TNFRSF). It acts as a costimulatory receptor on T cells, but its role on NK cells is poorly understood. CD137, another TNFRSF member has been shown to enhance the anti-tumour activity of NK cells in various malignancies. Here, we examine the expression and function of CD134 on human and mouse NK cells in B-cell lymphoma. CD134 was transiently upregulated upon activation of NK cells in both species. In contrast to CD137, induction of CD134 on human NK cells was dependent on close proximity to, or cell-to-cell contact with, monocytes or T cells. Stimulation with an agonistic anti-CD134 mAb but not CD134 ligand, increased IFNγ production and cytotoxicity of human NK cells, but this was dependent on simultaneous antibody:Fcγ receptor binding. In complementary murine studies, intravenous inoculation with BCL_1_ lymphoma into immunocompetent syngeneic mice resulted in transient upregulation of CD134 on NK cells. Combination treatment with anti-CD20 and anti-CD134 mAb produced a synergistic effect with durable remissions. This therapeutic benefit was abrogated by NK cell depletion and in Fcγ chain −/− mice. Hence, anti-CD134 agonists may enhance NK-mediated anti-tumour activity in an Fcγ receptor dependent fashion.

## Introduction

CD134 is a type I transmembrane glycoprotein that is transiently expressed on activated T cells, NK cells, NKT cells and neutrophils (reviewed in^1,2^) Its expression pattern is similar in both humans and mice, with the exception that CD134 is expressed constitutively on regulatory T cells (Tregs) in mice, but only upon activation on human Tregs^1^. Its function has been best characterised on CD4^+^ T cells where it acts as a co-stimulatory receptor. Engagement of CD134 by its ligand CD134L (CD252) or agonistic monoclonal antibodies (mAb) leads to recruitment of adaptor proteins called TNF associated factors (TRAFs) and stimulation of NFkB^3,4^, PI3K/PKB^5^ and NFAT pathways^6^ leading to increased survival, cell proliferation and cytokine production.

The anti-tumour efficacy of CD134 agonists in tumour models is variable and model-dependent. CD134 agonists alone have modest anti-tumour effects^7,8^, and are routinely used in combination with other agents to show efficacy e.g. with CpG and anti-CTLA-4^9^, with anti-HER2 and CTLA-4^10^, or with GITR stimulation^11^. The anti-tumour activity has been attributed to intratumoural Treg depletion or inactivation^9,12^ and CD4 and/or CD8 stimulation^7,10,13^. In the only reported clinical trial of anti-CD134 (which employed a mAb with a murine IgG1 isotype), tumour regressions were observed in patients with advanced cancer. Transient expansion of effector CD4^+^, CD8^+^ T and NK cells and increased vaccinal and tumour-specific T cell responses were also observed in some of the patients^14^.

In contrast to the wealth of data on T cells, there is a lack of understanding of the role of CD134 in NK cells. CD134 is reported to be expressed on NK cells^1^ but the requirements and kinetics of expression have not been characterised. Liu *et al*.^15^ previously described that engagement of CD134 on NK cells by CD134 ligand-expressing plasmacytoid dendritic cells resulted in the release of IFNγ in CD134L-deficient mice. However, this did not exclude the possibility of reverse signalling via CD134L itself^1^. Better understanding of the characteristics and function of CD134 on NK cells is highly relevant given the increasing number of anti-CD134 mAbs entering clinical development^16^, and the current efforts to exploit the anti-tumour efficacy of NK cells^17^.

CD137, another TNFRSF member was previously shown to be expressed on activated NK cells. Engagement of CD137 enhanced the therapeutic efficacy of direct-targeting mAb; rituximab (anti-CD20)^18^, trastuzumab (anti-HER2)^19^ and cetuximab (anti-EGFR)^20^ in B-cell lymphoma, breast and head and neck cancer models respectively.

The aim of the current investigation was to characterise the factors required for upregulation of CD134 on NK cells and determine if engagement would similarly enhance NK function. We demonstrate that CD134 is upregulated on activated NK cells in mice and humans. In humans, the upregulation of CD134 on NK cells is transient and requires close contact with activated T cells or monocytes. Stimulation of CD134 with an agonistic mAb enhanced the therapeutic efficacy of anti-CD20 treatment in a B-cell lymphoma mouse model and in an NK-dependent fashion. Engagement of CD134 on human NK cells with an agonistic mAb increased ADCC capacity, IFNγ secretion and showed a trend towards greater TNFα release. However, the observed functional effects on NK cells were strictly dependent on simultaneous Fcγ receptor (FcγR) cross-linking.

## Results

### Anti-CD134 augments anti-CD20 mAb-mediated immunotherapy of B-cell lymphoma

**Figure 1:**
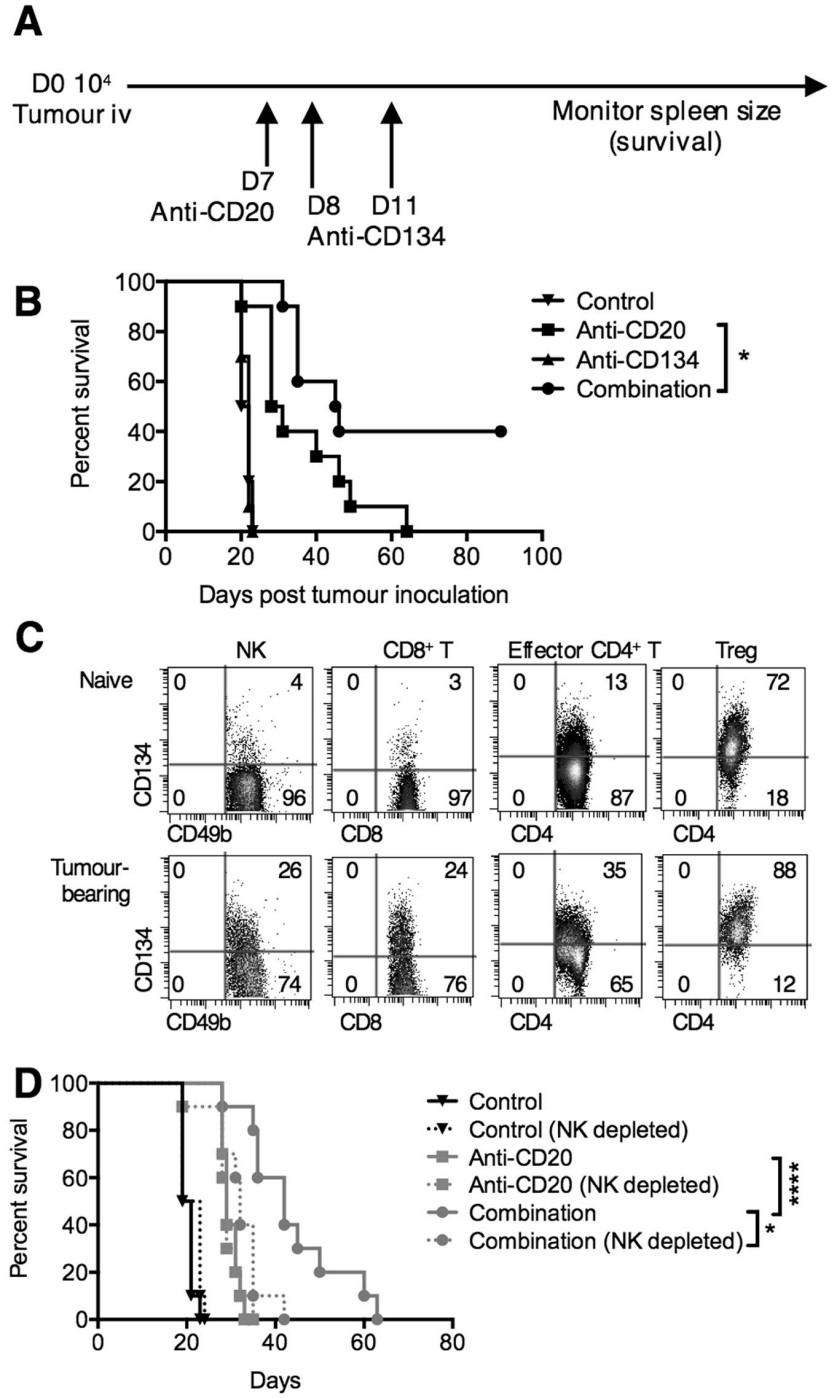
Anti-CD134 augments anti-CD20 mAb therapy in murine B-cell lymphoma. (**A**) BALB/c mice were inoculated i.v. with 10^4^ BCL_1_ cells on day 0. Mice were then treated with anti-CD20 (200 μg, i.p.) on day 7, and anti-CD134 (500 μg on days 8 and 11, i.p.). Tumour growth was monitored by splenic palpation and survival to the humane end-point plotted. (**B**) Kaplan-Meier survival curve of BCL_1_-bearing mice treated with either isotype control (control), anti-CD20, anti-CD134 or anti-CD20 and anti-CD134 (combination) as described in (**A**). n = 10/group, collated from two independent experiments. Log-rank (Mantel-Cox) test, *P < 0.01. (**C**) BALB/c mice were treated with PBS or 10^4^ BCL_1_ cells on day 0 and splenocytes harvested 24 hours later, and analysed by flow cytometry for CD134 expression on NK cells (CD3^−^NKp46^+^CD49b^+^), CD8^+^T cells (CD3^+^CD8^+^), effector CD4^+^ T cells (CD3^+^CD4^+^FOXP3^−^CD25^−^), or Treg (CD3^+^CD4^+^FOXP3^+^CD25^+^). The top row shows CD134 expression in naïve mice, and the bottom row, in BCL_1_-bearing mice. (**D**) BALB/c mice were treated with isotype control, anti-CD20 or anti-CD20 and anti-CD134 (combination) as described in (**A**). In addition, anti-ASGM1 (20 μL i.p.) was administered on days 4, 9, 14 and 19 to deplete NK cells. Kaplan Meier survival curves are shown. n = 10/group, collated from two independent experiments. Log-rank (Mantel-Cox) test, *P < 0.05, ****P < 0.0001.

To examine whether addition of agonistic anti-CD134 mAb^21,22^ improves the survival of mice treated with anti-CD20 mAb we employed the syngeneic B-cell lymphoma model, BCL_1_ (Fig. 1A). Here, tumour cells were injected intravenously (i.v.) on day 0 and treatment initiated sequentially with anti-CD20 on day 7 and anti-CD134 on days 8 and 11. Anti-CD134 alone did not impart any survival benefit (median survival 21 days and 22 days for control and anti-CD134 respectively) (Fig. 1B). Anti-CD20 treatment alone prolonged the survival of the mice to a median of 29.5 days but no mice survived beyond 62 days. The addition of anti-CD134 augmented the anti-CD20 therapy resulting in 40% of mice surviving beyond 100 days in some experiments. While long-term survivors were not always seen, consistently the combination outperformed either monotherapy (*P* < 0.05).

To provide insight into which cellular effectors were responsible for this augmentation we examined the expression of CD134 on lymphocyte subsets in naïve and BCL_1_-bearing mice (Fig. 1C). CD134 is not expressed on BCL_1_ cells (Supplementary Fig. 1), discounting direct cytotoxicity as a mechanism. In naïve mice, CD134 was expressed predominantly on Tregs, with a low level of expression on effector CD4^+^ T cells but not on NK or CD8^+^ T cells, consistent with previous observations (Fig. 1C). However, 24 hours post tumour inoculation, CD134 upregulation was observed on a proportion of NK cells and multiple T-cell subsets (NK: 26% positive, CD8^+^ T: 24%, effector CD4^+^ T: 35% and Treg: 88%). Administration of anti-CD20 did not affect the level of CD134 expressed on these cells (data not shown), similar to that observed with another tumour-targeting mAb in a xenograft mouse model^23^.

The contribution of NK cells to combined anti-CD20/anti-CD134 therapy was examined by NK cell depletion using anti-ASGM1^24^ (Fig. 1D). Depletion of the NK cells did not affect tumour growth or the efficacy of anti-CD20 alone. Although combination therapy did not confer long-term benefits in this experiment, nevertheless NK depletion reduced the therapeutic benefit of the combination to the level seen with single-agent anti-CD20 (median survival 42 days vs 32 days in undepleted vs NK depleted arms of combination therapy). Thus the beneficial effect of combined therapy is dependent on NK cells.

### Human NK cells ex press CD134 on co-culture with anti-CD20-coated targets

**Figure 2:**
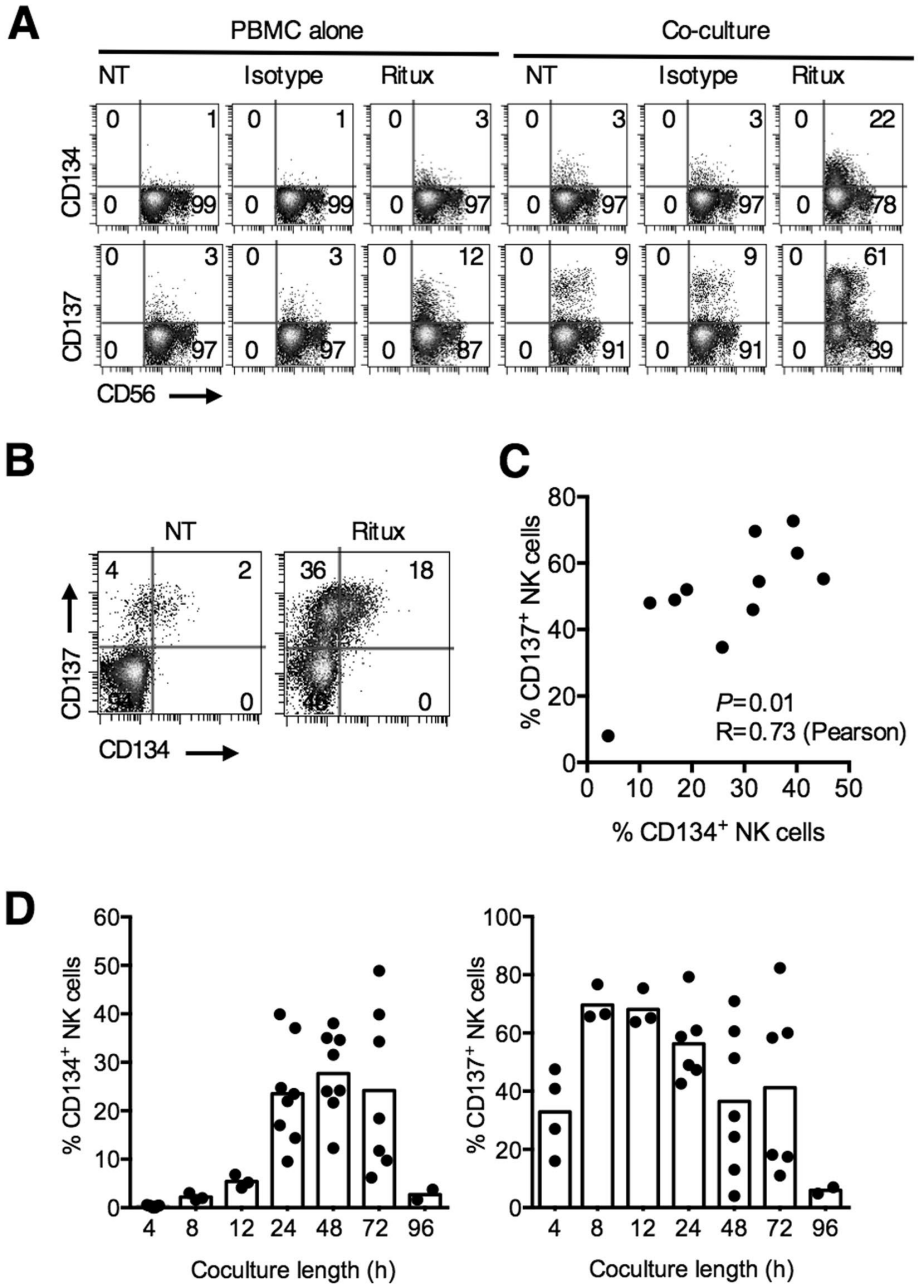
Human NK cells express CD134 on co-culture with anti-CD20-opsonised targets. (**A**) PBMCs from healthy donors were co-cultured with Ramos cells and not treated (NT) or treated with rituximab (5 μg/mL) or an isotype control for 24 hours. CD134 and CD137 expression on NK cells (CD56^+^CD3^−^) were examined by flow cytometry. Plots are representative of three independent experiments. (**B**) Dot plots from experiments performed as in (**A**) showing the expression of CD134 and CD137 on NK cells. Dot plots are representative of more than 6 experiments. (**C**) Cumulative data from (**B**) of concurrent CD134 and CD137 expression on NK cells are plotted and correlation performed using Pearson’s test. Each point representatives a different individual. (**D**) PBMCs were co-cultured with Ramos cells and rituximab for varying lengths of time, as indicated in the graph, harvested and examined by flow cytometry as in (**A**).

Next we examined whether CD134 was upregulated on human NK cells in the presence of mAb-opsonised tumour cells. We previously demonstrated that CD137 is upregulated on NK cells after co-culture with rituximab-opsonised B-cells^18^. Similarly, normal PBMCs co-cultured with rituximab-opsonised Ramos B-cell lymphoma cells resulted in the upregulation of CD134 on NK cells, albeit to a lesser extent compared to CD137 (22% vs 61% positive NK cells respectively) (Fig. 2A). Upregulation of CD134 (and CD137) was noted primarily on CD56^dim^ NK cells. In these cultures, NK cells that upregulated CD134 also co-expressed CD137 (Fig. 2B) with a direct correlation between CD134 and CD137 expression at 24 hours (Pearson’s correlation 0.73, *P* = 0.01) (Fig. 2C). Kinetically, upregulation of CD134 was however slower, with maximal expression (median 28% CD134^+^ NK cells) occurring 48 hours after co-culture, compared to 8 hours with CD137 (median 67% CD137^+^ NK cells) (Fig. 2D). Both CD134 and CD137 upregulation were transient, with expression lost 96 hours after co-culture.

### Upregulation of CD134 on human NK cells is dependent on T cells and/or monocytes

**Figure 3:**
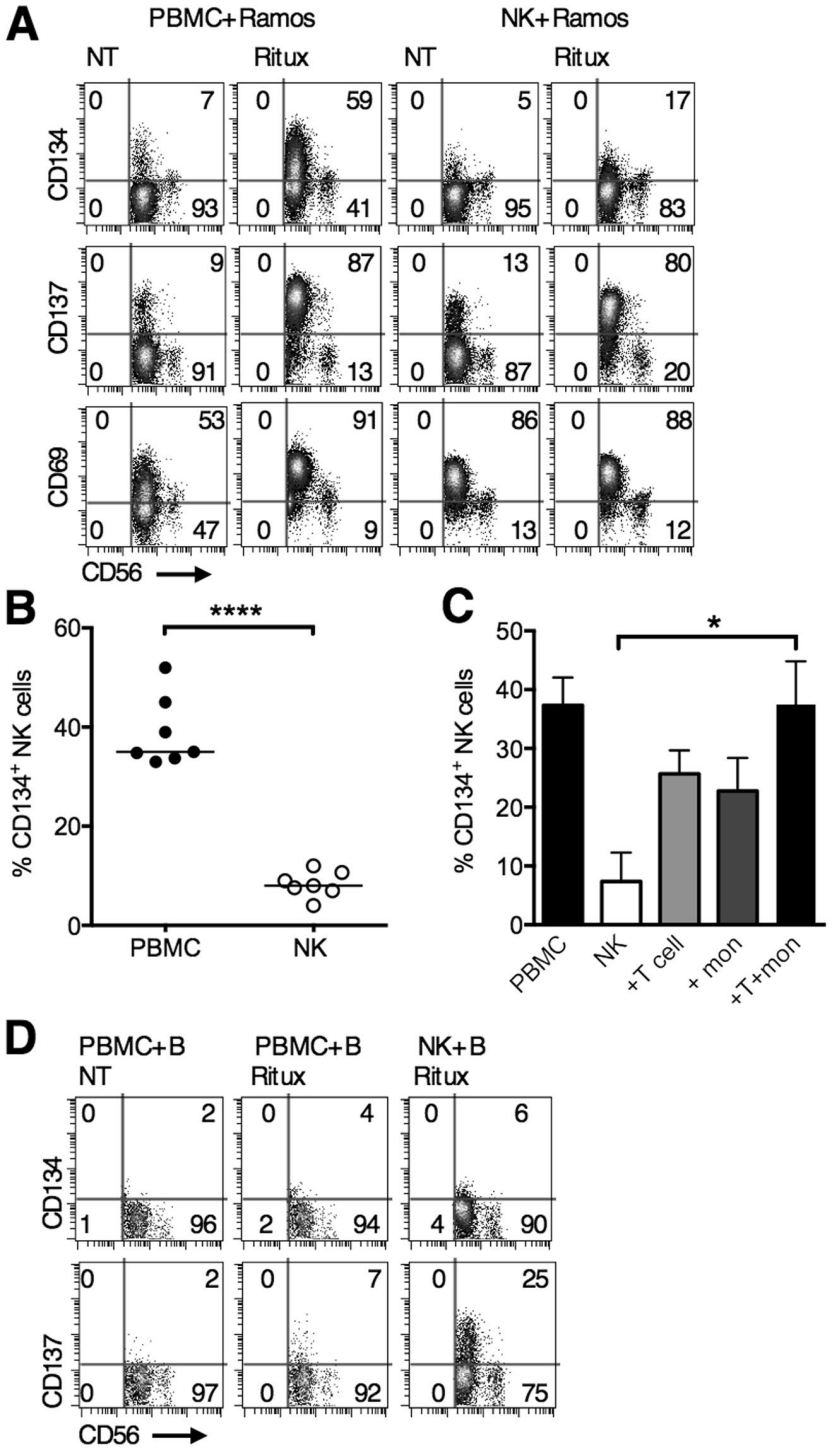
Expression of CD134 on human NK cells is dependent on interaction with T cells and/or monocytes. (**A**) PBMCs or purified NK cells were co-cultured with Ramos cells and not treated or treated with rituximab (5 μg/mL) for 24 hours. CD134, CD137 and CD69 expression on NK cells were then examined by flow cytometry. Representative plots shown from triplicate experiments. (**B**) Increase of %CD134 expression on NK cells within PBMC, and purified NK co-cultures, treated as in (**A**). The baseline %CD134 expression has been deducted. Each dot represents a different donor, ****P < 0.0001, paired t test. (**C**) PBMCs or purified NK cells treated with SEB and autologous purified T cells and/or purified monocytes added back. After 24 hours, co-cultures were examined by flow cytometry for CD134 expression on NK cells. n = 3 independent experiments, paired t test, *P < 0.05 (**D**) PBMCs or purified NK cells were co-cultured with autologous purified B-cells and not treated or treated with rituximab (5 μg/mL) for 24 hours. Co-cultures were then harvested and analysed by flow cytometry for CD134 and CD137 expression.

To explore if the upregulation was a direct effect, NK cells were isolated and co-cultured with rituximab-opsonised Ramos cells and upregulation of CD134, CD137 and CD69 examined 24 hours later (Fig. 3A). CD137 and CD69 upregulation occurred on NK cells equivalently whether purified NK cells or PBMCs were used. In contrast, CD134 upregulation occurred to a greater extent in PBMC co-cultures compared to purified NK co-cultures (median 35 vs 8 CD134^+^ NK cells, Fig. 3B). To evaluate the requirement of other cell types in upregulating CD134 on NK cells, T cells, monocytes, or T cells and monocytes together, were added back to purified NK cells in rituximab-opsonised Ramos cell co-cultures. Purified NK cells expressed low levels of CD134 (median 12.93%) but this was increased on addition of T cells, monocytes, or T cells and monocytes (T + mon) (24%, 24% and 40%, respectively) (Fig. 3C), thereby indicating that upregulation of CD134 in the co-cultures was dependent on the presence of T cells and/or monocytes. These responses were generated using opsonised allogeneic B-cells and so we subsequently examined whether rituximab-coated autologous B-cells would also upregulate CD134 on NK cells. In an autologous setting, all responses were markedly weaker: 25% of NK cells in these PBMC cultures upregulated CD137 (compared with 80% in PBMC allogeneic cultures) and 6% CD134 upregulation was seen (compared with 35% in PBMC allogeneic cultures). Responses to autologous B-cells with pure NK cells were weaker still (Fig. 3D). Altogether this suggested that the upregulation of CD134 on NK cells was in part dependent on the presence of allo-activated T cells or monocytes in the co-culture.

### CD134 is upregulated on human NK cells in the presence of activated T cells or monocytes

**Figure 4:**
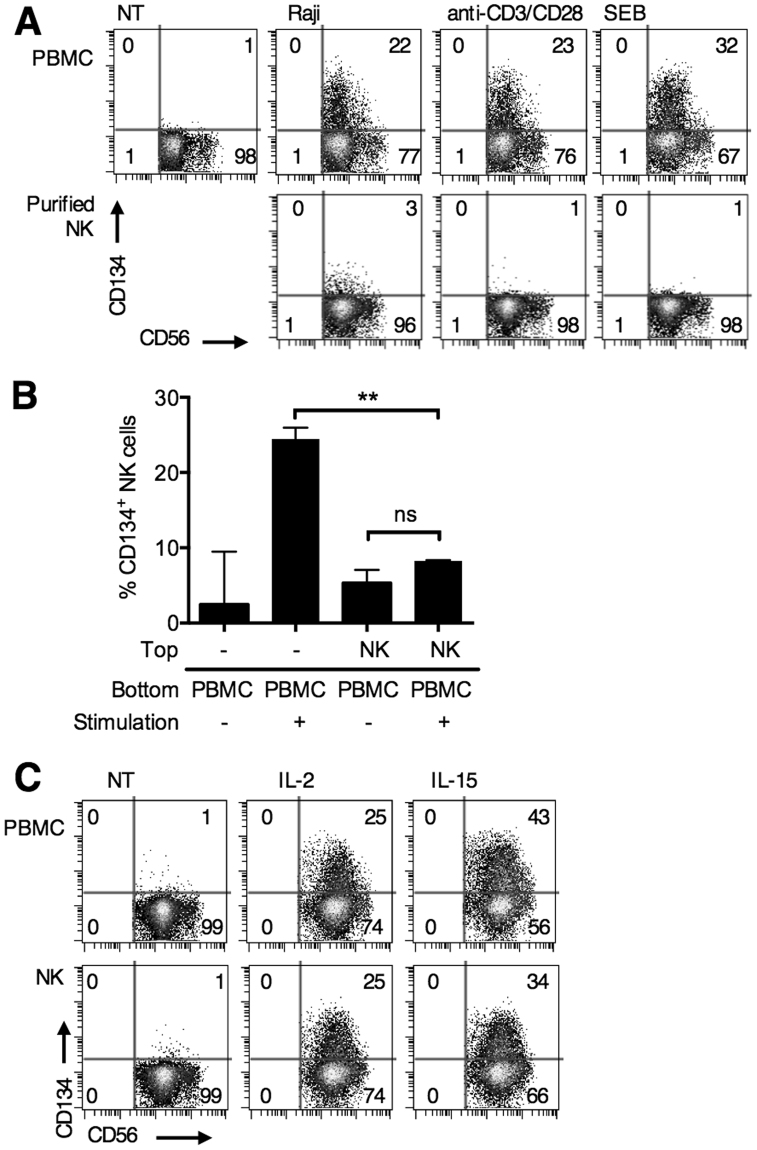
CD134 is upregulated on human NK cells in the presence of activated T cells. (**A**) PBMCs or purified NK cells were not treated, co-cultured with Raji cells, or treated with anti-CD3 (0.5 μg/mL) and anti-CD28 (1 μg/mL), or SEB (1 ng/mL) to stimulate T cells for 24 hours. CD134 expression was then examined by flow cytometry. CD134 expression on gated NK cells are shown. Plots representative of triplicate experiments. (**B**) PBMCs were unstimulated, stimulated with rituximab-treated B-cell targets, or stimulated and co-cultured in the same well with purified NK cells in a transwell plate. Co-cultures were harvested 24 hours later and CD134 expression on NK cells examined. n = 3 independent experiments, **P < 0.01, paired t test. (**C**) PBMCs or purified NK cells were unstimulated or treated with IL-2 (25 ng/mL) or IL-15 (10 ng/mL) for 24 hours. Cells were then harvested and analysed by flow cytometry. Representative dot plots shown from triplicate experiments.

To validate our hypothesis that activated T cells are required, we stimulated T cells in PBMC co-cultures using Raji cells (allogeneic stimulus), anti-CD3 and anti-CD28 mAb, or staphylococcal enterotoxin B (SEB, autologous stimuli) and then examined CD134 expression on NK cells (Fig. 4A). None of these T-cell stimulants upregulated CD134 on NK cells directly, as demonstrated when purified NK cells were used. However, within PBMC cultures, all three T-cell stimulants upregulated CD134 on NK cells (22%, 23% and 32% CD134^+^ NK cells using Raji, anti-CD3 and anti-CD28, and SEB respectively); clearly demonstrating the ability of activated T cells to induce CD134 expression on NK cells without the need to opsonise B-cells with anti-CD20 mAb.

Next we examined whether the NK cells required cell-to-cell contact with activated T cells/monocytes to upregulate CD134. To do this PBMCs were separated from purified NK cells in a transwell, and then stimulated using SEB (Fig. 4B). NK cells in close contact with T cells and monocytes (within PBMCs) upregulated CD134 (median 24.1% CD134^+^ NK cells), but the isolated NK cells present in the top of the transwell did not (median 8.3% CD134^+^ NK cells). This result indicates that NK cells either require cell-cell contact, or need to be in close proximity to T cells and monocytes, presumably where concentrations of cytokines are higher, to upregulate CD134.

To test whether prototypical cytokines (reviewed in^25^) produced by activated T cells (IL-2) and monocytes (IL-15) could elicit CD134 upregulation on NK cells, they were added to PBMC cultures or purified NK cells and CD134 upregulation examined (Fig. 4C). Upregulation of CD134 on NK cells was similarly observed in both scenarios.

### Agonistic anti-CD134 mAb promotes NK function in an Fc:FcγR dependent manner

**Figure 5:**
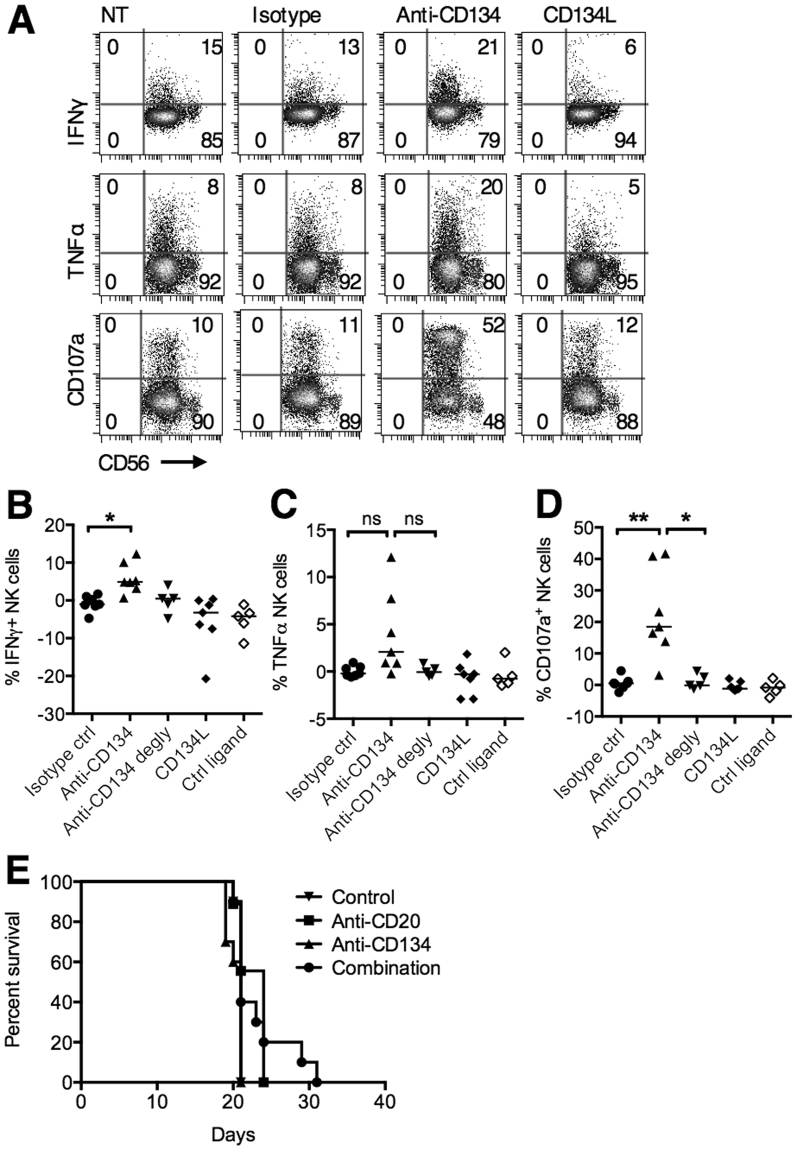
Agonistic anti-CD134 mAb promote NK associated cytokine release in an Fc:FcγR dependent manner. (**A**) PBMCs were stimulated with SEB (1 ng/mL) for 24 hours to upregulate CD134 expression on NK cells and then incubated with an isotype control, CD134 agonist mAb (5 μg/mL), or multimeric CD134 ligand (1 μg/mL) for 6 hours in the presence of brefeldin A or monensin. Intracellular IFNγ and TNFα, and surface CD107a expression were examined on NK cells by flow cytometry. Representative plots are shown from at least 5 experiments. (**B**–**D**) PBMCs were treated as described in (**A**). In addition, deglycosylated agonistic CD134 mAb and a multimeric control ligand was used. The graphs show the percentages of IFNγ^+^ (**B**), TNFα^+^ (**C**) and CD107a^+^ (**D**) NK cells after deduction of baseline expression on untreated cells. Paired t test, n = 5–6, *P < 0.05, **P < 0.01, ns = not significant. (**E**) BCL_1_-bearing Fcγ chain −/− mice were treated as in Fig. 1A. A Kaplan Meier survival curve is shown. n = 5/group, representative of duplicate experiments.

Having established the likely mechanism of CD134 upregulation on NK cells, we next examined its potential therapeutic function. Human PBMCs were first stimulated to upregulate CD134 on NK cells and then an agonistic anti-CD134 mAb (Supplementary Fig. 2) or a multimeric ligand for CD134 (CD134L) (Supplementary Fig. 3) was added for 6 hours before assessment of NK cell cytokine release and cytotoxicity (Fig. 5A). Agonistic anti-CD134 mAb increased release of IFNγ, triggered CD107a surface expression (a surrogate marker for NK cytotoxicity) and showed a trend towards increased TNFα expression, but the ligand had no effect. This suggested that CD134 engagement and signalling is insufficient for augmented effector functions and that simultaneous engagement of FcγR is required. The assays were therefore repeated using deglycosylated anti-CD134 (Supplementary Fig. 4A) which reduces binding to the high affinity FcγR, CD64 by 20-fold (data not shown) and abrogates binding to low affinity FcγRs (Supplementary Fig. 4B), whilst still preserving binding to CD134 (Supplementary Fig. 4C). Here, the mAb failed to elicit IFNγ, TNFα or CD107a expression, validating the requirement for simultaneous FcγR binding (Fig. 5B–D). Similarly, when a F(ab′)_2_ fragment of anti-CD134, was employed, upregulation of CD107a was not observed (Supplementary Fig. 5). Accordingly, we hypothesised that concurrent Fc:FcγR cross-linking is also required for the anti-CD134-mediated augmentation of anti-CD20 therapy in mice. Fcγ chain −/− mice bearing BCL_1_ lymphoma were treated with anti-CD20, anti-CD134, or the combination (Fig. 5E). In this setting, the therapeutic effect of combined anti-CD20 and anti-CD134 was lost, confirming the importance of activatory FcγRs in mediating the therapeutic effects.

## Discussion

With the success of checkpoint inhibitors in various solid malignancies, there is currently intense interest in the potential of cancer immunotherapy and the desire to exploit further immunomodulatory receptors (reviewed in^26^). CD134 is one of the targets of interest, with at least 6 different types of CD134 agonists in clinical trials^27^. Despite this, there is a complete lack of evidence on the importance of CD134 on NK cells. Our data demonstrates that CD134 is transiently expressed on NK cells upon activation, dependent on the presence of activated T cells or monocytes. Stimulation of NK cells with IL-2 or IL-15 also upregulates CD134, indicating that these cytokines may be the mediators of these effects from T cells and monocytes respectively. Engagement of CD134 with an agonistic mAb enhances NK cytotoxicity and cytokine effector function in an Fc: FcγR dependent fashion.

Our data demonstrates that in an immunocompetent, syngeneic lymphoma mouse model, CD134 is upregulated transiently on both T and NK cells post tumour inoculation. Anti-CD134 alone did not demonstrate any therapeutic effect, but when combined with anti-CD20, durable responses were observed in a proportion of mice. Depletion of NK cells did not change tumour growth in untreated mice, or affect the anti-CD20-treated arm, but reduced therapy of the combination arm such that it returned to the level of anti-CD20 therapy alone. It is important to recognise that durable responses were not demonstrated in all combination treated mice or in all experiments which might be due to inter-experimental variations in BCL_1_ given that this is an *in vivo* passaged tumour. Further, as this is an immunocompetent model, variations in immune response might also occur as a result of subtle differences in environmental stimuli beyond our control. Irrespective, there remains a statistically significant difference between the NK cell-depleted and non-depleted arms, and the combination arm was always superior to anti-CD20 alone.

In both mouse and human systems, CD134 is expressed to a lower degree than CD137, as shown here and in previous work^18^. Our human NK data show that in the human *in vitro* co-culture system, all CD134^+^ NK cells co-express CD137, but that only a proportion of CD137^hi^ NK cells co-express CD134. This suggests that the threshold for CD134 upregulation on NK cells are higher and that whilst both CD134 and CD137 are TNFRSF members, the pathways leading to activation may differ. The relatively low expression of CD134 on NK cells themselves might account for the lower enhancement of NK function on CD134 engagement in the mouse and *in vitro* compared to CD137, albeit different models are employed in the previously published CD137 experiments. Furthermore, the requirements for CD134 upregulation are clearly different from CD137. In the autologous human PBMC and B-cell co-cultures, CD137 but not CD134 was upregulated on NK cells. The upregulation of CD134 was specifically dependent on the presence of activated T cells and/or monocytes. In the tumour microenvironment of human cancers, CD134 is reported on CD4^+^ T cells^28,29^, but its expression on NK cells has not been defined. Taking into account the data seen here, it might be that CD134 may be more highly expressed on NK cells in more immunogenic tumours where activated T cells are present.

When purified NK cells were separated from PBMCs by a permeable membrane, minimal CD134 upregulation was observed on the purified NK cells, indicating that cell-to-cell contact is required. However, addition of exogenous IL-2 or IL-15 was sufficient to upregulate CD134 on NK cells, which seems at odds with the initial requirement for cell-to-cell contact. As proteins secreted at the cell surface are diluted with increasing distance^30^, it might be that actual cell-to-cell contact is not required, but that the NK cells have to be in close proximity to the cytokine secreting cell, in this case an IL-2 or IL-15 secreting T cell or monocyte respectively.

Stimulation of CD134 increased NK cytotoxicity and IFNγ expression, but only in the context of simultaneous FcγR cross-linking. Anti-CD134 hIgG1 increased CD107a and IFNγ expression, but not when the mAb was deglycosylated to abrogate FcγR binding. It is well-recognised that TNF receptors like CD134 and CD137 require receptor multimerisation for activation, and that Fc:FcγR interaction of an agonistic mAb helps to achieve this. However, when multimeric mouse CD134L^31^, capable of binding, multimerising and activating human CD134, was employed, NK degranulation and cytokine release was not seen. This suggests that the observed effects may be occurring through the NK Fc receptor, FcγRIII (CD16), itself capable of mediating cytotoxicity and cytokine release^32^. Seemingly against this hypothesis is the observation that the hIgG1 isotype control did not elicit any functional effects. However, it is important to recognise that FcγRIII is a low affinity receptor so effective stimulation requires Ab complexes or additional cross-linking^33^, which is provided by the binding of the F(ab) regions of the mAb to CD134. Blocking of FcγRIII by a mAb would further confirm this, but this approach is complicated by the fact that these mAb can also alter NK function, even as F(ab)_2_ fragments^32^.

In conclusion, our studies demonstrate that CD134 can be upregulated on NK cells in the presence of specific local environmental stimuli both in syngeneic, immunocompetent mouse models and human *in vitro* PBMC systems. It remains to be seen whether these observations apply in the human tumour microenvironment. Engagement of CD134 by agonistic mAb can stimulate NK cells to release IFNγ and increase ADCC, but this effect is entirely dependent on simultaneous engagement of FcγR cross-linking and might be mediated through the FcγR itself. These data highlight an important caveat in studies of agonistic NK receptor mAb, where an intact Fc domain of the mAb can stimulate FcγRIII on NK cells when simultaneous F(ab) and Fc cross-linking is present.

## Methods

### Mice

8-12 week old female BALB/c were supplied by Charles River Laboratories and maintained in the local animal research facility. Fcγ chain −/− mice have been previously described^34^. Animal experiments were approved by the University of Southampton Ethical Committee and conducted according to UK Home Office license guidelines.

### Tumour model

The murine B-cell lymphoma cell line, BCL_1_ was maintained by passage in BALB/c mice^35^. For the BCL_1_ tumour model, 1 × 10^4^ tumour cells were injected i.v. into the tail veins of BALB/c mice, and treatment initiated with anti-CD20 (18B12 mIgG2a, 200 μg, i.p., produced in-house)^36^ on day 7 and anti-CD134 (OX86 rIgG1, 500 μg i.p., produced in-house)^21,22^ on days 8 and 11. Tumour growth was monitored by palpation of the spleen and mice were culled humanely before reaching terminal endpoint. NK cell depletion was carried out using anti-ASGM1 Ab (Biolegend)^24^. Anti-ASGM1 20 μL i.p. was administered on days 4, 9, 14 and 19 and depletion efficiency inspected by flow cytometry in the blood and spleen.

### Human cells and PBMC preparation

PBMCs were obtained from anonymised leukocyte cones of healthy donors via the National Blood Service (Southampton, UK) and density gradient centrifugation performed (Lymphoprep). Human sample use was approved by the East of Scotland Research Ethics Service, in line with the Declaration of Helsinki. Informed consent was obtained from all participants.

### Human NK cell stimulation assays

PBMCs or purified NK cells were stimulated through various means. For the co-culture assay, PBMCs or NK cells were cultured with B-cell targets at 1–2 × 10^6^/mL for 24 hours. Alternatively PBMCs or NK cells were cultured with anti-CD3 (OKT3, 0.5 μg/mL, in house) and anti-CD28 (CD28.2, 1 μg/mL, eBioscience), recombinant human IL-2 (25 ng/mL, Biotechne), recombinant human IL-15 (10 ng/mL, Biotechne) or SEB (Sigma) (1 ng/mL) for 24 hours.

### Flow cytometry

Human single cell suspensions were surface stained with fluorescence conjugated mAb to CD134 (L106, BD Bioscience), CD137 (4B4-1, BD Bioscience), CD69 (FN50, eBioscience), CD56 (N901, Beckman Coulter), CD3 (SK7, eBioscience) and CD5 (OKT1, in house). Murine cells were first FcγR-blocked (2.4G2, in-house) and then stained with CD49b (DX5, eBioscience), NKp46 (29A1.4, eBioscience), CD3 (17A2, eBioscience), CD4 (RM4–5, eBioscience), CD8 (53–6.7, eBioscience) or CD25 (PC61.5, eBioscience). For intracellular FOXP3 (NRRF-30, eBioscience) staining, cells were fixed and permeabilised as per manufacturer’s protocol (eBioscience). Acquisition was performed using FACSCalibur (BD Biosciences) and analysed using Cytobank (Cytobank).

### Human CD134 stimulation assays

Stimulated PBMCs were further co-cultured with an isotype control (cetuximab, Southampton General Hospital pharmacy), anti-CD134 (SAP25–29 h1, in-house), deglycosylated anti-CD134 (in-house), anti-CD134 F(ab′)_2_ (in-house, produced as previously described^37^), multimeric CD134L (Caltag) or control ligand (Caltag) for 6 hours. For IFNγ (B27, BD Bioscience) and TNFα (Mab11, eBioscience) intracellular staining, human cells were fixed and permeabilised as per manufacturer’s protocol (BD Bioscience) after 6 hours co-culture with brefeldin A (BD Bioscience). For assessment of NK degranulation, PBMCs were incubated with CD107a-PE (H4A3, BD Bioscience) and monensin (BD Bioscience) for 6 hours.

### Protein deglycosylation

Anti-huCD134 mAb was deglycosylated using N-Glycosidase F (PNGase F) (Promega), as per manufacturer’s protocol. Briefly, the mAb was incubated with the PNGase F at 37 °C for 48 hours and then deglycosylation inspected by gel-shift on SDS-PAGE.

### Cell isolation

T cell, NK cell, B-cell and monocytes were isolated by negative selection using magnetic-activated cell sorting kits (Miltenyi Biotec).

### Transwell assay

1 × 10^6^ PBMCs were cultured in the bottom wells, and purified NK cells in the top well (2 × 10^5^) of a 48-well plate with 0.4 μm membrane (Corning) and unstimulated, or stimulated with SEB for 24 hours. Wells were individually harvested and washed before staining for CD134 expression on NK cells.

### Statistical analysis

Data are shown as medians +/− ranges. Data were analysed using student’s t-test (two-tailed). The log-rank (Mantel-Cox) test was used to analyse differences between Kaplan Meier survival curves. In all analyses, *P* < 0.05 was considered significant.

### Data availability

No datasets were generated or analysed during the current study.

## Electronic supplementary material

Supplementary Information

## References

[1] Croft M (2010). Control of immunity by the TNFR-related molecule OX40 (CD134). Ann Rev Immunol..

[2] Willoughby J, Griffiths J, Tews I, Cragg MS (2017). OX40: Structure and function - What questions remain?. Mol Immunol..

[3] Kawamata S, Hori T, Imura A, Takaori-Kondo A, Uchiyama T (1998). Activation of OX40 signal transduction pathways leads to tumor necrosis factor receptor-associated factor (TRAF) 2- and TRAF5-mediated NF-kappaB activation. J Biol Chem..

[4] Arch RH, Thompson CB (1998). 4-1BB and Ox40 are members of a tumor necrosis factor (TNF)-nerve growth factor receptor subfamily that bind TNF receptor-associated factors and activate nuclear factor kappaB. Mol Cellular Biol..

[5] Song J (2004). The costimulation-regulated duration of PKB activation controls T cell longevity. Nat Immunol..

[6] So T, Song J, Sugie K, Altman A, Croft M (2006). Signals from OX40 regulate nuclear factor of activated T cells c1 and T cell helper 2 lineage commitment. Proc Natl Acad of Sci USA.

[7] Kjaergaard J (2000). Therapeutic efficacy of OX-40 receptor antibody depends on tumor immunogenicity and anatomic site of tumor growth. Cancer Res..

[8] Weinberg AD (2000). Engagement of the OX-40 receptor *in vivo* enhances antitumor immunity. J Immunol..

[9] Marabelle A (2013). Depleting tumor-specific Tregs at a single site eradicates disseminated tumors. J Clin Invest..

[10] Linch SN (2016). Combination OX40 agonism/CTLA-4 blockade with HER2 vaccination reverses T-cell anergy and promotes survival in tumor-bearing mice. Proc Natl Acad of Sci USA.

[11] Leyland R (2017). A Novel Murine GITR Ligand Fusion Protein Induces Antitumor Activity as a Monotherapy That Is Further Enhanced in Combination with an OX40 Agonist. Clin Cancer Res..

[12] Piconese S, Valzasina B, Colombo MP (2008). OX40 triggering blocks suppression by regulatory T cells and facilitates tumor rejection. J Exp Med..

[13] Gough MJ (2008). OX40 agonist therapy enhances CD8 infiltration and decreases immune suppression in the tumor. Cancer Res..

[14] Curti BD (2013). OX40 is a potent immune-stimulating target in late-stage cancer patients. Cancer Res..

[15] Liu C (2008). Plasmacytoid dendritic cells induce NK cell-dependent, tumor antigen-specific T cell cross-priming and tumor regression in mice. J Clin Invest..

[16] Linch SN, McNamara MJ, Redmond WL (2015). OX40 Agonists and Combination Immunotherapy: Putting the Pedal to the Metal. Frontiers Oncol..

[17] Guillerey C, Huntington ND, Smyth MJ (2016). Targeting natural killer cells in cancer immunotherapy. Nature Immunol..

[18] Kohrt HE (2011). CD137 stimulation enhances the antilymphoma activity of anti-CD20 antibodies. Blood.

[19] Kohrt HE (2012). Stimulation of natural killer cells with a CD137-specific antibody enhances trastuzumab efficacy in xenotransplant models of breast cancer. J Clin Invest..

[20] Kohrt HE (2014). Targeting CD137 enhances the efficacy of cetuximab. J Clin Invest..

[21] al-Shamkhani A (1996). OX40 is differentially expressed on activated rat and mouse T cells and is the sole receptor for the OX40 ligand. Eur J Immunol..

[22] Taraban VY (2002). Expression and costimulatory effects of the TNF receptor superfamily members CD134 (OX40) and CD137 (4-1BB), and their role in the generation of anti-tumor immune responses. Eur J Immunol..

[23] Bezman NA (2017). PD-1 blockade enhances elotuzumab efficacy in mouse tumor models. Blood Advances.

[24] Turaj AH, Dahal LN, Beers SA, Cragg MS, Lim SH (2017). TLR-3/9 Agonists Synergize with Anti-ErbB2 mAb-Letter. Cancer Res..

[25] Floros T, Tarhini AA (2015). Anticancer Cytokines: Biology and Clinical Effects of Interferon-alpha2, Interleukin (IL)-2, IL-15, IL-21, and IL-12. Seminars Oncol..

[26] Mellman I, Coukos G, Dranoff G (2011). Cancer immunotherapy comes of age. Nature.

[27] US National Institutes of Health. *ClinicalTrials*.*gov*. https://clinicaltrials.gov (2017).

[28] Vetto JT (1997). Presence of the T-cell activation marker OX-40 on tumor infiltrating lymphocytes and draining lymph node cells from patients with melanoma and head and neck cancers. Am J Surg..

[29] Sarff M (2008). OX40 (CD134) expression in sentinel lymph nodes correlates with prognostic features of primary melanomas. Am J Surg..

[30] Gurdon JB, Bourillot PY (2001). Morphogen gradient interpretation. Nature.

[31] Mestas J, Crampton SP, Hori T, Hughes CC (2005). Endothelial cell co-stimulation through OX40 augments and prolongs T cell cytokine synthesis by stabilization of cytokine mRNA. Int Immunol..

[32] Galatiuc C (1995). Natural killer (NK) activity in human responders and nonresponders to stimulation by anti-CD16 antibodies. Cellular Immunol..

[33] Nimmerjahn F, Ravetch JV (2008). Fcgamma receptors as regulators of immune responses. Nature Rev Immunol..

[34] Beers SA (2010). Antigenic modulation limits the efficacy of anti-CD20 antibodies: implications for antibody selection. Blood.

[35] Slavin S, Strober S (1978). Spontaneous murine B-cell leukaemia. Nature.

[36] Williams EL (2013). Immunotherapy targeting inhibitory Fcgamma receptor IIB (CD32b) in the mouse is limited by monoclonal antibody consumption and receptor internalization. J Immunol..

[37] Lim SH (2011). Fc gamma receptor IIb on target B cells promotes rituximab internalization and reduces clinical efficacy. Blood.

