# Supplementary material for: Augmentation of CD134 (OX40)-dependent NK anti-tumour activity is dependent on antibody cross-linking
Source: Sci Rep. 2018 Feb 2;8:2278. doi: 10.1038/s41598-018-20656-y (PMC5797108; doi:10.1038/s41598-018-20656-y)
Supplement: Supplementary file 1 — Supplementary Information [file 41598_2018_20656_MOESM1_ESM.pdf]

Supplementary Material

Augmentation of CD134 (OX40)-dependent NK anti-tumor activity is dependent on antibody cross-linking

Anna H Turaj, Kerry L Cox, Christine A Penfold, Ruth R French, C Ian Mockridge, Jane Willoughby, Alison L Tutt, Jordana Griffiths, Peter W M Johnson, Martin J Glennie, Ronald Levy, Mark S Cragg, and Sean H Lim

Supplementary Methods

T-cell proliferation assays

Fresh PBMCs were labelled with 1 μM carboxyfluorescein succinimidyl ester (CFSE) and cultured at high density (1×10^7/mL) for 48 hours prior to antibody stimulations. For the PBMC stimulation, cells were transferred into round-bottomed 96-well plates at 1×10^5 per well and treated with OKT3 (as specified in the Figure Legends) and either 1 μg/ml (soluble) multimeric CD134L (Caltag) or 5 μg/ml anti-huCD134 mAb, SAP25-29. Proliferation was assessed by CFSE dilution and CD8 or CD4 staining on a FACSCanto II flow cytometer (BD Biosciences).

Surface plasmon resonance analysis

SPR analysis was performed on a Biacore T200 (GE Healthcare). Samples were run at 25°C in HBS-EP+ buffer at 30 μL/min. Data was analysed with BiaEvaluation software. The response of the blank control flow-cell was automatically subtracted prior to data analysis. For comparison of human CD134 binding, 100 RU of huCD134-hFc (R&D Systems) was immobilised at pH5 onto a CM5 sensor chip by amine coupling and serial antibody dilutions

(threefold from 500 nM) were injected across the surface for 300s. Dissociation was monitored for 600s.

For comparison of FcR binding, 5000RU of WT and deglycosylated anti-huCD134 were serially immobilised onto a CM5 sensor chip at pH5 by amine coupling. 100nM of FcγR (R&D Systems) were injected across both surfaces for 180s.

Supplementary Figure Legends

Supplementary Figure 1. CD134 is not expressed on BCL_1 cells. BCL_1 cells from a terminal mouse were harvested, FcγR blocked, and stained for BCL_1 idiotype, CD19 and CD134 and analysed by flow cytometry. The histogram overlay shows the CD134 and isotype control staining of BCL_1 cells. Histograms and MFI values are representative of 3 independent experiments.

Supplementary Figure 2. The anti-huCD134 mAb, SAP25-29 is agonistic (**A**) 293F cells were transiently transfected with a WT huCD134 construct 24 hours prior to flow cytometry analysis. Isotype control (anti-huCD137, mIgG1) or anti-huCD134 (mIgG1) mAb were added to 100 μl of cells at a final concentration of 10μg/ml for 15 minutes at RT before washing and detection with a secondary PE-labelled anti-mouse $F(ab)_2$ fragment. (**B**) PBMCs were labelled with CFSE and then cultured at high density for 2 days in 10% RPMI. Cells were harvested, and stimulated with 0.75 μg/ml OKT3 and 5 μg/ml SAP25-29 for 5 days. Cells were then labelled with anti-CD8 APC and division assessed by flow cytometry. Representative plots are shown. (**C**) The graph shows the cumulative data from (B). Each point represents a different individual. ***P*<0.01

Supplementary Figure 3. Mouse multimeric CD134L stimulates human CD134. PBMCs were labelled with CFSE and then cultured at high density for 2 days. Cells were

then harvested and stimulated with 1 μg/ml OKT3 and 1 μg/ml multimeric CD134L. On day 6, CD4 T cell proliferation assessed by CFSE dilution and % of cells that have undergone more than 1 division calculated and plotted from triplicate wells. Data from 2 independent donors. * $P<0.05$, ** $P<0.01$

Supplementary Figure 4. Anti-huCD134 deglycosylation. (**A**) The SDS-PAGE gel shows the non-reduced and reduced native, and deglycosylated anti-huCD134 mAbs. (**B**) SPR analysis of native and deglycosylated anti-huCD134 binding to the low affinity human FcγRs indicated. (**C**) SPR analysis of native and deglycosylated anti-huCD134 binding to huCD134-Fc at the concentrations indicated.

Supplementary Figure 5. Anti-huCD134 F(ab')$_2$ does not activate NK cells. PBMCs were stimulated with SEB as described in Fig. 5, and then incubated with an isotype control, anti-CD134 IgG1 or anti-huCD134 F(ab')$_2$ (all 5 μg/mL) for 6 hours in the presence of monensin and CD107a-PE, and examined by flow cytometry. The graph shows the percentage of CD107a$^+$ cells after deduction of baseline expression on untreated cells.

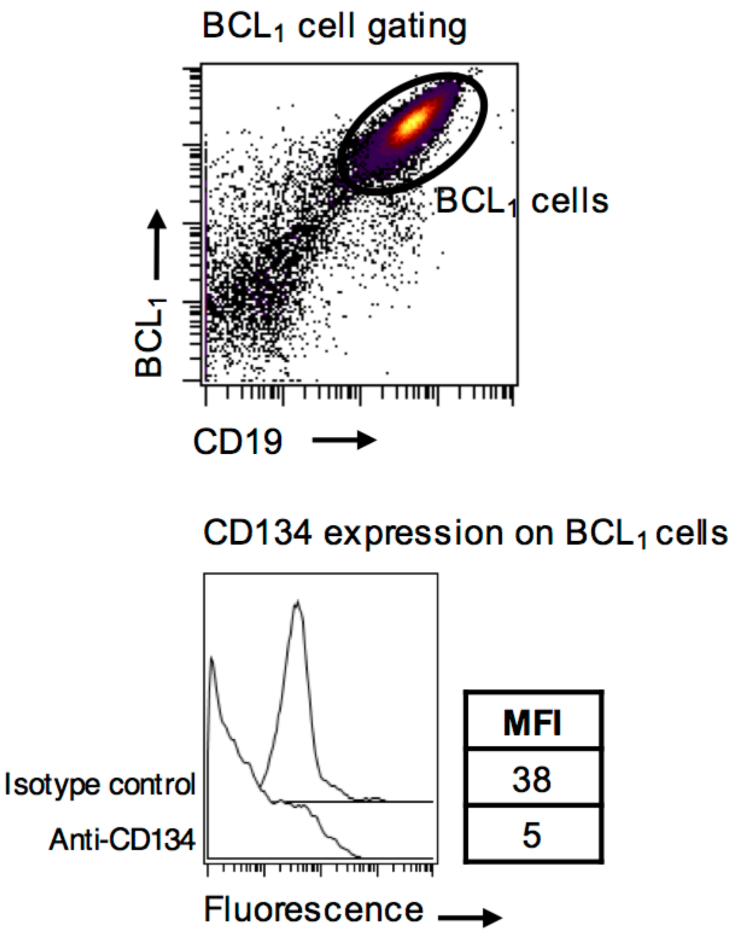

Supplementary Figure 1

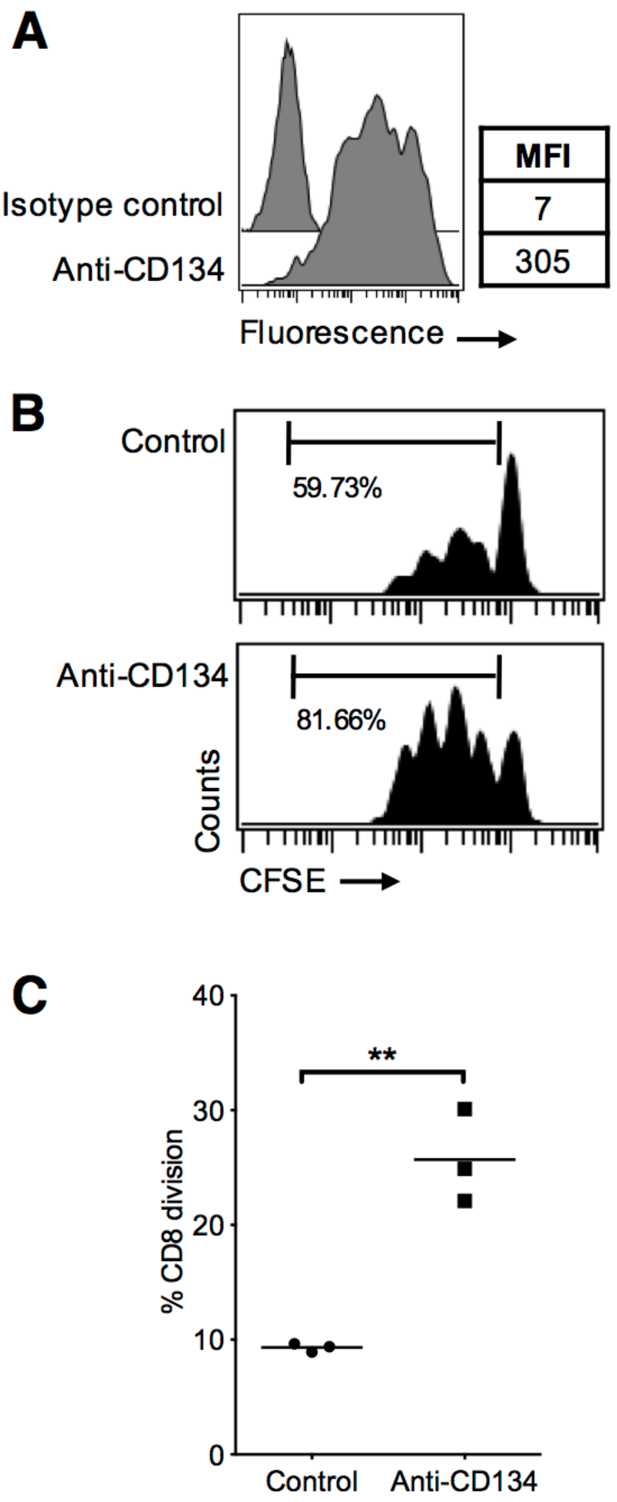

Supplementary Figure 2

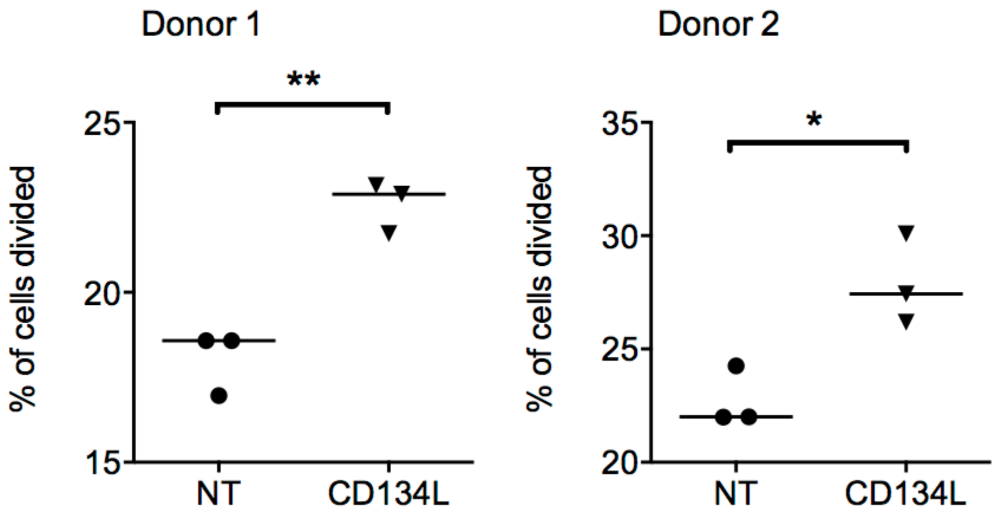

Supplementary Figure 3

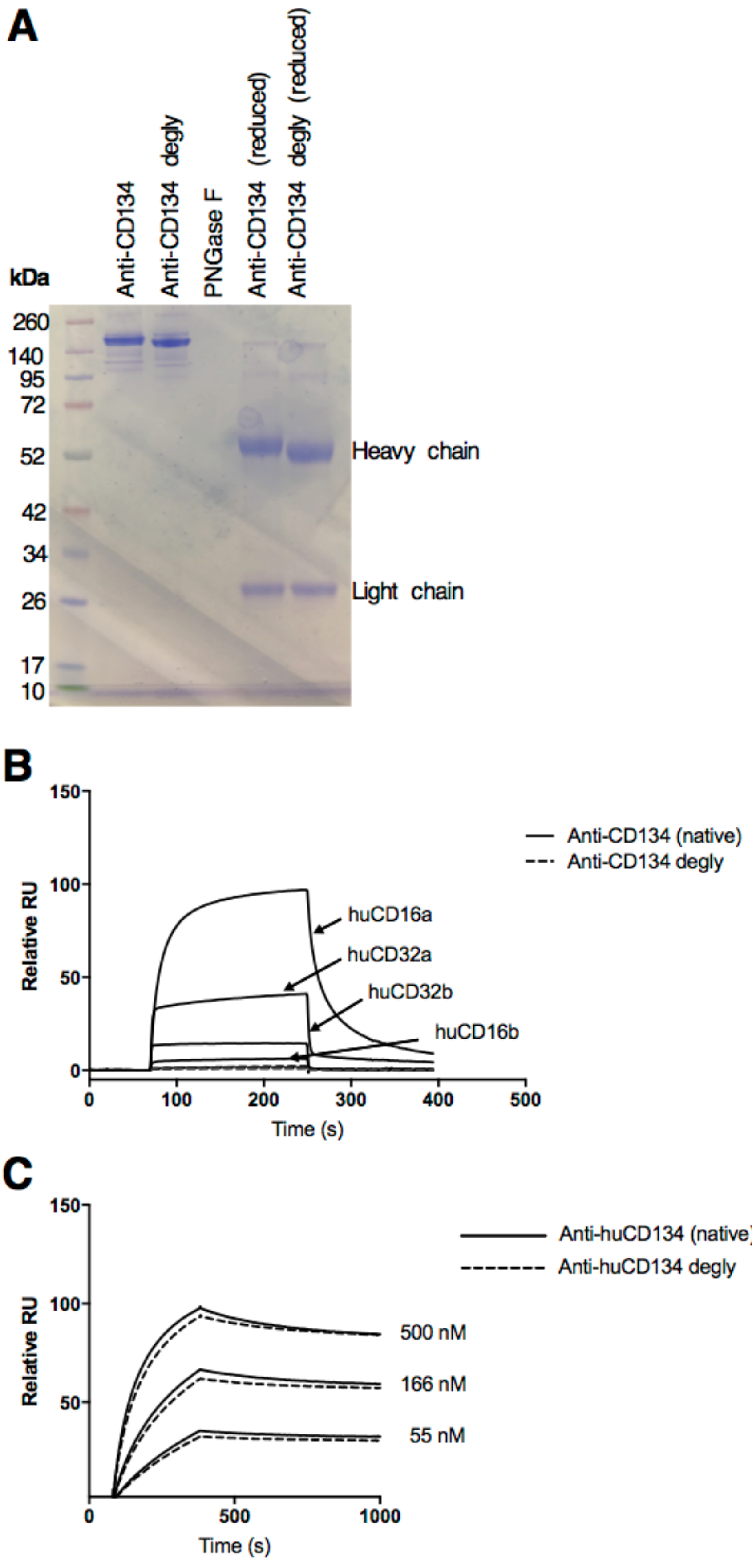

Supplementary Figure 4

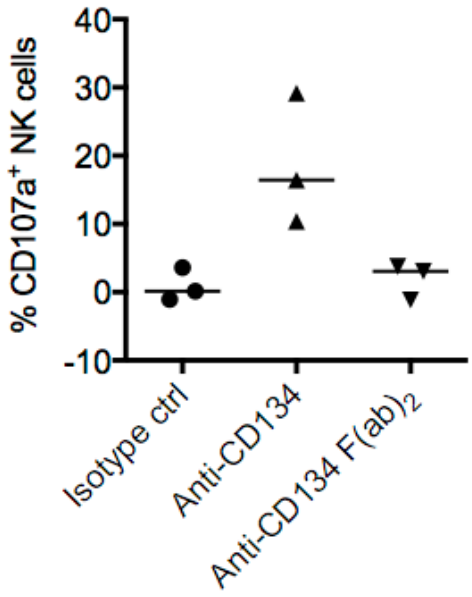

Supplementary Figure 5
